# Cytokines as Potential Biomarkers of Clinical Characteristics of Schizophrenia

**DOI:** 10.3390/life12121972

**Published:** 2022-11-25

**Authors:** Irina A. Mednova, Anastasiia S. Boiko, Elena G. Kornetova, Arkadiy V. Semke, Nikolay A. Bokhan, Svetlana A. Ivanova

**Affiliations:** 1 Mental Health Research Institute, Tomsk National Research Medical Center, Russian Academy of Sciences, Aleutskaya Str. 4, Tomsk 634014, Russia; 2 Hospital at Siberian State Medical University, Moskovsky Trakt 2, Tomsk 634050, Russia; 3 Department of Psychiatry, Addictology and Psychotherapy, Siberian State Medical University, Moskovsky Trakt 2, Tomsk 634050, Russia

**Keywords:** cytokine, schizophrenia, biomarker, clinical feature

## Abstract

Immune activation plays a major role in the pathogenesis of schizophrenia, as confirmed by many studies, systematic reviews, and meta-analyses. The important role of neuroinflammation in the formation of the relation between impaired neurobiological processes and schizophrenia psychopathology is being actively discussed. We quantified serum concentrations of 22 cytokines in 236 patients with schizophrenia and 103 mentally and somatically healthy individuals by a multiplex assay. We found higher TGF-α (*p* = 0.014), IFN-γ (*p* = 0.036), IL-5 (*p* < 0.001), IL-6 (*p* = 0.047), IL-8 (*p* = 0.005), IL-10 (*p* <0.001), IL-15 (*p* = 0.007), IL-1RA (*p* = 0.007), and TNF-α (*p* < 0.001) levels in patients with schizophrenia than in healthy individuals. Subgroup analysis revealed a much greater number of statistically significant differences in cytokine levels among females than among males. Patients with a continuous course of schizophrenia showed statistically significantly higher levels of IL-12p70 (*p* = 0.019), IL-1α (*p* = 0.046), and IL-1β (*p* = 0.035) compared with patients with an episodic course. Most cytokines were positively correlated with positive, general, and total PANSS scores. In patients with a duration of schizophrenia of 10 years or more, the level of IL-10 was higher than that in patients with a disease duration of 5 years or less (*p* = 0.042). Thus, an imbalance in cytokines was revealed in patients with schizophrenia, depending on sex and clinical characteristics of the disease.

## 1. Introduction

Schizophrenia is a chronic, heterogeneous, progressive, multifactorial mental disorder of unknown etiology and pathogenesis characterized by positive (e.g., delusions and hallucinations), negative (e.g., anhedonia and social withdrawal), and affective symptoms, as well as cognitive dysfunction. It has been suggested that environmental factors adversely interact with genetic predisposition, thus leading to the development of schizophrenia spectrum disorders [1]. Dopamine, serotonin, and glutamate hypotheses of psychosis have become classic theories of schizophrenia [2]. In recent years, due to the accumulation of a large amount of information, the hypothesis of immune dysregulation in schizophrenia has come onto the scene [3,4,5,6,7,8]. A large study on 36,989 schizophrenia patients and 113,075 controls has revealed associations with genes that play an important role in immunity [9]. A comparative review of serum/plasma biomarkers that can be used to differentiate schizophrenia from healthy individuals indicates that more than 70% of potential markers are involved in the inflammatory response [10]. Uncontrolled activation of proinflammatory cytokines and microglia, along with genetic variants of glutamatergic neurotransmission, may be some of the pathogenetic mechanisms of schizophrenia [11]. Randomized placebo-controlled trials have been conducted to evaluate the effectiveness of adjunctive use of anti-inflammatory drugs in schizophrenia and have yielded inconclusive results [12,13]. A meta-analysis has shown the ability of nonsteroidal anti-inflammatory drugs (celecoxib or acetylsalicylic acid) to reduce the severity of schizophrenia as compared with a placebo, according to PANSS. Nonetheless, those authors explain that these results should be interpreted with caution because these are first-time studies, and the sample size is small [14]. The involvement of the immune system in the pathophysiology of schizophrenia is supported by the following findings: (a) an imbalance between pro- and anti-inflammatory cytokines and an effect of antipsychotic medication on their levels [15,16,17,18]; (b) activation of microglia, as confirmed by animal studies, postmortem brain studies, and positron emission tomography [19,20,21]; (c) higher prevalence of autoantibodies [22,23]; (d) activation of inflammation-related genes in the brain [24,25]; and (e) increased expression of proinflammatory genes in circulating monocytes [26]. Peripheral cytokines can cross the blood–brain barrier in patients with schizophrenia after damage but also bind to specific transporters, penetrate via afferent vagal fibers, and get access through circumventricular organs [27,28]. In addition, it is reported that microglia, astrocytes, neurons, and endothelial cells can produce cytokines [28,29,30]. According to meta-analyses, there is an increase in levels of proinflammatory cytokines in schizophrenia [31,32]. Nevertheless, data on specific cytokines vary. For example, Wei et al. [33] have found no significant changes in the levels of IL-6 in schizophrenia patients; low TNF-α levels in schizophrenia were found to correlate with negative symptoms and in chronic schizophrenia with long-term use of antipsychotics [34,35]. Furthermore, there is no significant increase in IL-1β levels in chronic patients [36], and there are diminished IL-1β and TNF-α levels in first-episode schizophrenia with a disease duration of less than 2 years [37]. Results about T-helper and T-regulatory cytokines are even more inconsistent [32,38]. The discrepancies in the cytokine profile among the studies may be due to various factors: differences in patient characteristics owing to the high heterogeneity of schizophrenia, small sample sizes, differences in the tested parameters between men and women, and not taking into consideration metabolic disorders in the study population. Therefore, there is a need for new research taking into account the possible relationship of clinical features of schizophrenia with serum levels of cytokines.

Previously, we have demonstrated the effect of atypical antipsychotics on peripheral cytokine levels as well as the role of cytokines in the formation of metabolic syndrome in schizophrenia [39,40]. We have also examined the influence of changes in glutamatergic neurotransmission on the onset and course of schizophrenia [41]. Via the promotion of oxidative stress, there is considerable commonality between glutamatergic excitotoxicity and inflammatory damage [5,6,8]. It is, therefore, a logical step to also investigate the relationship between cytokine levels and clinical characteristics of schizophrenia. We hypothesized that patients with schizophrenia have an imbalance between pro- and anti-inflammatory cytokines, and we tried to detect possible correlations between clinical characteristics and the presumed cytokine imbalance.

## 2. Materials and Methods

### 2.1. Participants

After signing a voluntary informed consent, 236 patients with schizophrenia (F20 according to the International Classification of Diseases-10 [ICD-10], predominantly with paranoid schizophrenia [F20.0]) were enrolled in the study. The control group consisted of 103 mentally and somatically healthy individuals who provided written informed consent. The control group comes from the same geographical area as the patients with schizophrenia and included mainly students and employees of the Siberian State Medical University (Tomsk, Russia) and Tomsk National Research Medical Center. The age range of the patients and healthy individuals eligible for the study was 18–60 years. Patients with schizophrenia and healthy individuals who had signs of acute or chronic infectious, inflammatory, or autoimmune diseases at the time of enrollment in the study were not allowed to participate in the study. The study did not include persons with comorbid neurological and somatic diseases, which make it difficult to objectively assess the clinical condition and persons who used psychoactive substances (other than antipsychotic medication).

The severity of schizophrenia symptoms was determined using the Positive and Negative Syndrome Scale (PANSS) [42]. This scale consists of a subscale of 7 items assessing the severity of positive symptoms of schizophrenia, a negative subscale of 7 items evaluating symptoms caused by loss of skill, and a subscale of 16 items about general psychiatric symptoms, such as anxiety, tension, and disorientation.

The clinical course of schizophrenia was determined in accordance with ICD-10 criteria (F20.x1–3 for an episodic course and F20.x0 for a continuous course). Mean doses of antipsychotic medication used were converted to chlorpromazine equivalents [43].

### 2.2. Laboratory Tests

Blood samples were drawn after a 12-h overnight fast on the first days of hospitalization and centrifuged for 30 min at 2000× *g* and 4 °C to isolate serum; the serum was stored at −80 °C until analysis. The concentrations of 22 cytokines (IL-1α, IL-1β, IL-2, IL-3, IL-5, IL-6, IL-7, IL-8, IL-9, IL-12p40, IL-12p70, IL-15, IL-17A, IFN-α2, IFN-γ, TNF-α, TNF-β, IL-1RA, IL-4, IL-10, IL-13, and TGF-α) in blood serum were determined using the HCYTMAG-60K-PX41 panel of MILLIPLEX^®^ MAP (Merck, Darmstadt, Germany) on multiplex analyzer MAGPIX and Luminex 200 (Luminex, Austin, TX, USA) at the Medical Genomics core facility of Tomsk National Research Medical Center. The obtained data were processed in special software, xPONENT (Luminex, Austin, TX, USA), and the output was exported to the MILLIPLEX Analyst 5.1 software (Merck, Darmstadt, Germany). The final results on cytokine concentrations are presented in pg/mL.

### 2.3. Statistics

Statistical analysis was performed in SPSS software (version 20) for Windows. We used several methods of group comparisons, such as the Shapiro–Wilk test, the Mann–Whitney *U* test, the Kruskal–Wallis test, and the χ^2^ test. For multiple comparisons, the Bonferroni correction was applied. Correlation analysis was performed by means of Spearman’s criteria. Data with *p*-values of less than 0.05 were considered statistically significant.

## 3. Results

### 3.1. The Main Demographic and Clinical Characteristics of Participants

The study included 236 patients with schizophrenia and 103 healthy individuals. There were 121 (51.3%) females among patients with schizophrenia and 51 (53.4%) among the healthy subjects (*p* = 0.405). The median age (interquartile range) of patients with schizophrenia and healthy individuals was 36 (30; 46) and 35 (27.5; 46.5) years, respectively (*p* = 0.446). The median age of females with schizophrenia and healthy females was 36 (30; 44) and 42 (31; 52) years, respectively (*p* = 0.072), and the median age of males with schizophrenia and healthy males was 34 (29.5; 41) and 32 (26.5; 38.5) years, respectively (*p* = 0.065). The main demographic and clinical characteristics of the patients with schizophrenia are shown in Table 1.

### 3.2. Serum Concentrations of Cytokines in Patients with Schizophrenia

We found higher TGF-α (*p* = 0.014), IFN-γ (*p* = 0.036), IL-5 (*p* < 0.001), IL-6 (*p* = 0.047), IL-8 (*p* = 0.005), IL-10 (*p* < 0.001), IL-15 (*p* = 0.007), IL-1RA (*p* = 0.007), and TNF-α (*p* < 0.001) levels in patients with schizophrenia compared with healthy individuals (Table 2).

Subgroup analysis revealed a much greater number of statistically significant differences in cytokine levels among females than among males. For instance, in females with schizophrenia, there were statistically significantly greater concentrations of TGF-α (*p* = 0.006), IFN-γ (*p* = 0.005), IL-1β (*p* = 0.038), IL-10 (*p* < 0.001), IL-12p40 (*p* = 0.047), IL-15 (*p* = 0.001), IL-17A (*p* = 0.037), IL-1RA (*p* = 0.023), IL-5 (*p* = 0.006), IL-6 (*p* = 0.025), IL-8 (*p* = 0.004), and TNF-α (*p* < 0.001) and lower IL-3 levels (*p* = 0.046) compared with healthy age-matched females. Males with schizophrenia had statistically significantly higher levels of IL-10 (*p* = 0.017), IL-5 (*p* = 0.048), and TNF-α (*p* = 0.025) than did healthy age-matched males (Table 3).

### 3.3. Serum Concentrations of Cytokines and Clinical Characteristics of Schizophrenia

Patients with a continuous course of schizophrenia showed statistically significantly greater levels of IL-12p70 (*p* = 0.019), IL-1α (*p* = 0.046), and IL-1β (*p* = 0.035) compared to patients with an episodic course (Table 4).

**Figure 1:**
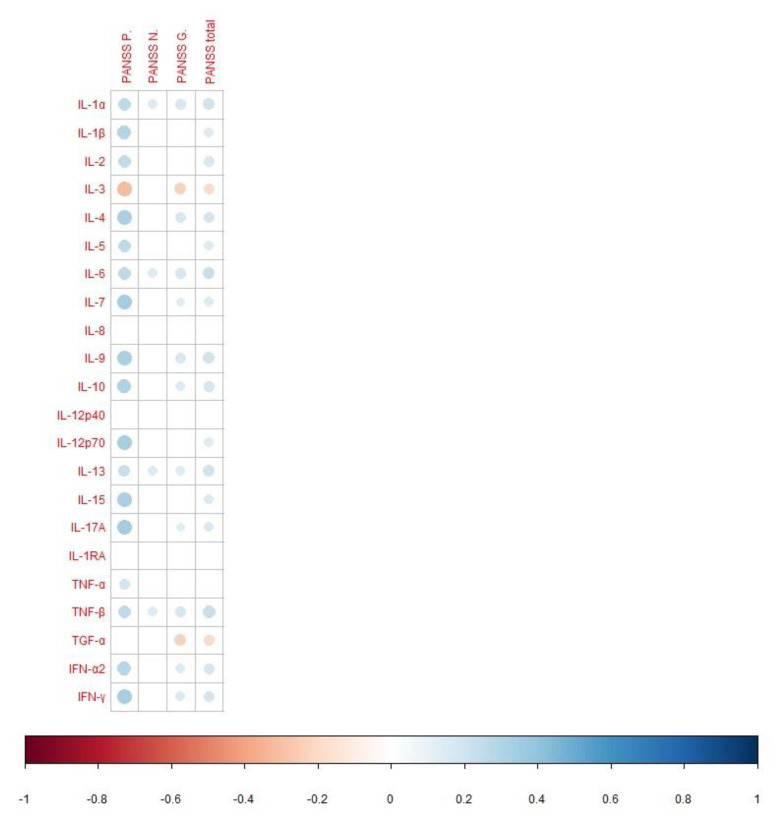
Spearman’s correlation analysis of the cytokines and disease severity (according to PANSS) in patients with schizophrenia. PANSS P.: the positive symptom score from PANSS; PANSS N.: the negative symptom score from PANSS; PANSS G.: the general psychopathology score from PANSS; PANSS total: the total PANSS score. In this figure, the red and blue circles refer to significant negative and positive correlations, respectively. The size and color intensity of the circles is proportional to the correlation coefficient. In the legend at the bottom, the intensity of the color shows the coefficients of correlation and their sign (inverse or direct correlation).

According to the correlation analysis, most cytokines are positively correlated with positive, general, and total PANSS scores. Only IL-8, IL-12p40, and IL-1RA did not statistically significantly correlate with the severity of schizophrenia, according to PANSS (Figure 1).

In patients with a duration of schizophrenia of 10 years or more, the level of IL-10 was higher than that in patients with a disease duration of 5 years or less (*p* = 0.042; Table S1). There were no statistically significant differences in the levels of cytokines between groups of the age of schizophrenia onset (Table S2).

## 4. Discussion

In this work, we found significantly higher serum levels of proinflammatory (IFN-γ, IL-5, IL-6, IL-8, IL-15, and TNF-α) and anti-inflammatory (TGF-α, IL-10, and IL-1RA) cytokines in patients with schizophrenia than in healthy individuals. Overall, the findings are consistent with other studies and meta-analyses about serum cytokines in schizophrenia [31,32]. A considerable number of studies, including meta-analyses, have revealed elevated levels of IL-6 and TNF-α in various groups of patients with schizophrenia [32]. Some of the best-known proinflammatory cytokines belong to the IL-1 family. We found no difference in IL-1α and IL-1β levels between the schizophrenia group and control group. According to Momtazmanesh et al. [32], the literature contains mixed data on blood levels of these cytokines in schizophrenia. IL-1RA competes with IL-1α and IL-1β for binding to the IL-1 receptor and competitively inhibits IL-1 activity [44]. Our findings about elevated serum levels of IL-1RA in patients with schizophrenia are consistent with those of Zhou et al., although they have not detected changes in concentrations of any other cytokines [45]. The increased IL-8 serum concentration in our study is in agreement with the results of a meta-analysis indicating an elevated concentration of this cytokine in chronic schizophrenia [16]. On the other hand, in patients with the first episode of schizophrenia, the level of IL-8 is comparable to the control [46]. Alterations in IFN-γ levels are controversial: several research groups have found no significant alterations of IFN-γ and IL-10 levels, whereas other studies show either increased or decreased levels of IFN-γ in patients with schizophrenia [32]. Elevated levels of IL-5 in the serum of patients with schizophrenia have been demonstrated [47], in line with our results.

Summarizing the above, we can conclude that there is considerable heterogeneity of data regarding blood levels of cytokines in patients with schizophrenia. As mentioned in the Introduction, these discrepancies may be due to the dissimilarity of clinical characteristics among studies, the use of antipsychotic medication, and/or insufficient sample sizes.

In terms of immune-system activation and neuroinflammation, which can influence symptoms and severity of the disease, schizophrenia may be similar to neurological and psychiatric diseases that also feature an imbalance in peripheral cytokines [15,48,49,50]. It is worth underscoring our finding that the aberration of the cytokine profile in schizophrenia is more pronounced in females than in males. In the general population, significant effects of sex and phase of the menstrual cycle on plasma concentration of cytokines have been demonstrated [51]. Some research on patients with schizophrenia indicates statistically significantly greater IL-1β, IL-8, IL-17, IL-23, and TNF-α levels in women but not in men, compared with healthy individuals [52]. Frydecka et al. [53] have shown that the *TGFB1* +869T/C gene polymorphism is associated with schizophrenia, especially among females; no differences were found in the severity of schizophrenia symptoms registered by PANSS between males and females with schizophrenia, suggesting that sex is a potentially important factor for group differences in cytokine levels. This phenomenon is probably related to the effects of sex hormones. According to in vivo studies, sex hormones can modulate cytokine production and contribute to sex-related differences in immune responses to health and disease [54,55]. This observation may serve as a theoretical basis for anti-inflammatory treatment of women with schizophrenia.

In our study, patients with a continuous course of schizophrenia had elevated levels of proinflammatory cytokines (IL-12p70, IL-1α, and IL-1β) as compared with patients with an episodic course. In a clinical sense, proinflammatory cytokines—as some of the pathogenesis components—play an important role in clinical polymorphism and the course of schizophrenia through reactivity mechanisms. Malashenkova et al. [56] have reported that episodic schizophrenia and continuous schizophrenia have signs of systemic inflammation. At the same time, the continuous course of schizophrenia is characterized mainly by chronic activation of the humoral response, whereas the episodic one by some activation of cell-mediated immunity. In another work, serum levels of soluble TNF-α receptor 1 were found to be higher in patients with a severe clinical course of schizophrenia [57]. Our study was conducted on a group of patients with a long duration of the illness, which can be considered a limitation of the study on the one hand. On the other hand, this work allows us to use the obtained results as predictors of schizophrenia course along with other determinants [58] because after 5 years of illness, the type of schizophrenia course is already evident [59].

Most of the assayed cytokines were positively correlated with positive, general, or total PANSS scores, thereby reflecting a direct link between immunoinflammation and schizophrenia severity. The fewest relations were observed between cytokine levels and negative symptoms of schizophrenia: only IL-1, IL-6, IL-13, and TNF-β were positively correlated with the negative-symptom PANSS score. Our data are partly consistent with the review by Momtazmanesh et al. [32], according to which there is a correlation between negative symptoms with elevated levels of IL-6, TNF-α, IL-1β, IL-8, IFN-γ, IL-4, and TGF-β. In addition, higher levels of IL-6, IL-1β, IL-33, and IL-17 are associated [32] with more severe positive symptoms. Increased levels of IL-6, IL-33, sIL-2R, IL-17, and TGF-β positively correlate with the general psychopathology subscore of PANSS [32]. The total PANSS score is positively associated with the levels of IL-6, sIL-2R, IL-1β, IFN-γ, IL-13, TGF-β1, and IL-17 [32]. Our findings are also partly consistent with a study that has uncovered correlations between proinflammatory cytokines and positive, general, and total PANSS scores but detected no correlation between cytokine levels and the negative-symptom PANSS score [60]. Our results are in agreement with articles showing correlations between IL-6 and PANSS scores among individuals with schizophrenia, with at-risk mental states, or with another psychotic disorder [61,62]. The link between the upregulation of proinflammatory cytokine IL-10 and ≥10-year duration of schizophrenia may be explained by the activation of compensatory mechanisms. Pedrini et al. [63] have also demonstrated elevated IL-10 levels in patients at ≥10 years after a diagnosis of schizophrenia as compared with healthy individuals. According to a meta-analysis [16], blood levels of IL-6 are significantly positively correlated with disease duration in three studies, although this result is not confirmed by another study. We, too, found no relation between IL-6 levels and schizophrenia duration.

The limitations of our study are as follows: the patients had long-duration schizophrenia, as mentioned above. Therefore, we cannot rule out the long-term impact of antipsychotic medication on the tested cytokines. Evaluation of the long-term effect of antipsychotic medication on the studied parameters was also not possible because the patients had been admitted to the hospital with severe clinical symptoms. Some of the patients were on maintenance treatment. We could glean information about the use of antipsychotics only from the patients’ own words and could not adequately control compliance and drug doses. We have previously evaluated the influence of short-term antipsychotic medication on serum cytokine levels [39].

When assessing the influence of sex-related differences on the levels of cytokines, we did not specify the menstrual cycle, which also plays an important part in the regulation of these parameters. Nonetheless, many women with schizophrenia experience menstrual irregularities, at least partly as an adverse effect of antipsychotic drugs.

From the findings, we can conclude that schizophrenia is characterized by a cytokine imbalance, and manifestations of this imbalance differ depending on sex, the severity of clinical symptoms, and disease duration. Research has shown that adjunctive treatment with acetylsalicylic acid may alleviate schizophrenia symptoms and reduce mortality from cardiovascular disease, which is common in schizophrenia [14]. Our study indirectly confirms the reasons for the heterogeneity of the clinical response to anti-inflammatory therapy in patients with schizophrenia. 

## 5. Conclusions

In patients with schizophrenia, we found a cytokine imbalance that correlates with sex and clinical characteristics of the disease. Although schizophrenia involves changes within the immune system, it is difficult to get a grip on the biological mechanisms involved. A deeper understanding of the disturbances of the cytokine network in schizophrenia may facilitate early diagnosis, prognosis, and personalized treatment.

## Figures and Tables

**Table 1:** Basic demographic and clinical characteristics of patients with schizophrenia included in the study.

Parameter		Total (*n* = 236)	Females (*n* = 121)	Males (*n* = 115)	*p*-Value
Age, years		36 (30; 46)	36 (30; 44)	34 (29.5; 41)	0.380
Age at disease manifestation, years		23 (19; 30)	24.5 (20; 31)	23 (19; 27)	0.115
Duration of disease, years		12 (5; 19)	11 (5; 18)	12 (5; 17)	0.956
Clinical course, *n* (%)	Episodic course	87 (49.7%)	52 (55.3%)	35 (43.2%)	0.179
	Continuous course	71 (40.6%)	32 (34.0%)	39 (48.1%)	
PANSS, positive score		21 (17; 25)	21 (17; 26)	21 (17; 25)	0.714
PANSS, negative score		25 (21; 28)	24 (21; 28)	26 (22; 28)	0.045
PANSS, general score		53 (44.5; 57.5)	53 (47; 58)	53 (44; 57)	0.379
PANSS, total score		100 (87.5; 109)	100 (87; 109)	100 (91; 109)	0.742
Duration of antipsychotic therapy, years		7 (3; 16)	7 (3; 15)	7 (3; 15.5)	0.744
Total CPZeq		350 (200; 623)	342.4 (200; 675)	387.54 (200; 600)	0.940

Note: Data are presented as a median (lower quartile; upper quartile); PANSS: Positive and Negative Syndrome Scale; CPZeq: chlorpromazine equivalents. Comparisons between groups were performed by the χ^2^ test for the clinical course and by the Mann–Whitney *U* test for the other parameters.

**Table 2:** Cytokine levels in the serum of patients with schizophrenia and healthy individuals.

Parameter	Patients with Schizophrenia	Healthy Individuals	*p*-Value
Proinflammatory cytokines			
IL-1α	70.83 (52.61; 105.10)	61.84 (53.98; 111.89)	0.660
IL-1β	3.25 (2.33; 4.48)	2.85 (1.86; 4.84)	0.110
IL-2	6.11 (4.68; 7.12)	5.90 (4.83; 8.10)	0.406
IL-3	2.55 (1.31; 3.32)	3.05 (1.38; 3.70)	0.116
IL-5	3.39 (1.98; 4.40)	2.04 (1.34; 3.86)	0.001 *
IL-6	10.55 (5.28; 15.48)	6.55 (4.60; 14.68)	0.047 *
IL-7	13.09 (9.72; 29.14)	14.14 (9.72 (29.55)	0.632
IL-8	12.87 (9.26; 20.03)	10.58 (7.19; 18.53)	0.005 *
IL-9	4.87 (3.32; 13.16)	4.36 (2.81; 14.45)	0.637
IL-12p40	45.83 (38.90; 52.70)	44.52 (32.37; 56.46)	0.233
IL-12p70	9.62 (6.74; 24.17)	8.32 (6.67; 22.12)	0.371
IL-15	9.05 (6.26; 11.71)	7.23 (4.57; 11.42)	0.007 *
IL-17A	6.58 (4.33; 14.85)	5.60 (3.74; 14.47)	0.152
IFN-α2	34.00 (17.32; 90.62)	30.57 (17.50; 87.69)	0.871
IFN-γ	14.80 (10.12; 23.01)	11.76 (8.44; 21.56)	0.036 *
TNF-α	22.87 (16.94; 28.93)	18.01 (9.76; 23.40)	<0.001 *
TNF-β	20.87 (6.01; 30.15)	7.80 (5.01; 33.81)	0.114
Anti-inflammatory cytokines			
IL-1RA	47.08 (38.55; 63.58)	44.79 (33.22; 52.85)	0.007 *
IL-4	111.61 (75.13; 159.29)	92.78 (71.53; 160.49)	0.351
IL-10	12.90 (8.00; 25.67)	7.93 (5.84; 20.56)	<0.001 *
IL-13	18.52 (13.18; 24.02)	17.47 (12.18; 27.70)	0.717
TGF-α	5.06 (4.00; 7.06)	4.61 (2.86; 7.20)	0.014 *

Note: Data are presented as a median (lower quartile; upper quartile); comparisons between groups were performed using the Mann–Whitney *U* test; *: statistically significant differences.

**Table 3:** Cytokine levels in the serum of patients with schizophrenia and healthy individuals depending on sex.

Parameter	Females			Males		
	Patients with Schizophrenia	Healthy Individuals	*p*-Value	Patients with Schizophrenia	Healthy Individuals	*p*-Value
Proinflammatory cytokines						
IL-1α	81.16 (54.66; 106.80)	59.21 (50.20; 94.87)	0.102	63.68 (52.36; 105.10)	64.84 (57.60; 111.89)	0.193
IL-1β	3.68 (2.39; 4.57)	2.71 (1.58; 4.22)	0.038 *	3.10 (2.33; 4.48)	2.85 (2.41; 4.85)	0.913
IL-2	6.21 (4.91; 7.12)	5.90 (4.82; 7.95)	0.907	5.63 (4.53; 7.35)	6.03 (4.85; 8.36)	0.230
IL-3	2.17 (1.23; 3.32)	3.20 (1.74; 3.69)	0.046 *	2.81 (1.32; 3.34)	2.91 (1.32; 3.70)	0.886
IL-5	3.79 (2.07; 4.53)	2.05 (1.27; 4.18)	0.006 *	3.08 (1.89; 4.27)	2.03 (1.38; 3.72)	0.048 *
IL-6	10.87 (6.23; 15.78)	6.55 (4.45; 14.93)	0.025 *	8.26 (4.99; 15.18)	6.31 (4.74; 13.58)	0.590
IL-7	15.84 (10.66; 29.91)	12.00 (9.69 (27.55)	0.245	11.35 (9.39; 28.35)	16.34 (10.64 (31.42)	0.070
IL-8	12.92 (9.44; 21.21)	8.88 (6.34; 18.53)	0.004 *	12.31 (8.99; 19.31)	11.48 (8.24; 18.48)	0.313
IL-9	7.77 (3.52; 13.16)	4.23 (2.54; 14.45)	0.328	4.61 (3.04; 12.30)	4.43 (2.81; 14.45)	0.717
IL-12p40	45.73 (38.90; 52.70)	42.10 (28.55; 49.27)	0.047 *	45.83 (38.90; 53.29)	46.29 (37.23; 57.83)	0.786
IL-12p70	15.68 (6.88; 24.63)	7.99 (6.15; 20.93)	0.099	8.42 (6.71; 23.71)	8.70 (7.12; 22.79)	0.645
IL-15	9.34 (6.26; 12.01)	6.44 (3.72; 10.24)	0.001 *	8.75 (6.26; 11.27)	8.07 (6.12; 12.30)	0.820
IL-17A	10.72 (4.29; 14.85)	4.61 (3.25; 15.96)	0.037 *	5.86 (4.39; 14.66)	7.73 (4.61; 12.98)	0.712
IFN-α2	43.38 (18.59; 90.62)	26.67 (15.99; 82.42)	0.140	28.89 (16.01; 86.21)	36.33 (23.90; 93.53)	0.084
IFN-γ	17.14 (10.00; 23.01)	9.65 (7.65; 25.88)	0.005 *	13.08 (10.12; 23.37)	13.00 (10.31; 21.04)	0.925
TNF-α	22.42 (16.57; 28.66)	15.27 (8.41; 21.29)	<0.001 *	23.02 (17.87; 28.93)	20.26 (12.51; 25.09)	0.025 *
TNF-β	23.20 (6.44; 30.15)	7.22 (3.98; 33.82)	0.090	11.92 (6.00; 29.23)	7.94 (6.39; 30.60)	0.879
Anti-inflammatory cytokines						
IL-1RA	50.23 (41.23; 67.74)	43.68 (32.26; 59.45)	0.023 *	44.96 (38.13; 57.94)	43.90 (36.21; 51.25)	0.176
IL-4	130.1 (81.48; 165.30)	90.57 (65.82; 145.09)	0.095	92.78 (72.14; 152.06)	95.09 (73.42; 171.82)	0.671
IL-10	18.94 (8.00; 26.41)	7.43 (5.58; 16.50)	<0.001 *	11.95 (7.77; 24.56)	9.04 (6.27; 19.30)	0.017 *
IL-13	19.47 (13.75; 24.02)	16.01 (10.14; 28.06)	0.122	16.35 (12.89; 24.02)	19.64 (12.97; 26.97)	0.270
TGF-α	5.15 (4.09; 7.16)	4.03 (2.51; 6.14)	0.006 *	4.96 (3.77; 7.06)	4.70 (3.01; 7.93)	0.456

Note: Data are presented as a median (lower quartile; upper quartile); groups were compared by the Mann–Whitney *U* test; *: statistically significant differences.

**Table 4:** Cytokine levels in the serum of patients with schizophrenia depending on the type of clinical course.

Parameter	Episodic Course (*n* = 87)	Continuous Course (*n* = 71)	*p*-Value
Proinflammatory cytokines			
IL-1α	74.27 (52.48; 105.1)	94.87 (56.95; 118.65)	0.046 *
IL-1β	3.69 (2.31; 4.48)	4.13 (2.94; 5.00)	0.035 *
IL-2	6.21 (5.01; 7.12)	6.67 (5.07; 7.57)	0.262
IL-3	2.1 (1.2; 3.34)	1.56 (1.26; 3.32)	0.780
IL-5	3.59 (2.02; 4.27)	4.13 (2.24; 4.67)	0.084
IL-6	11.18 (5.75; 17.27)	12.12 (6.87; 15.78)	0.788
IL-7	14.43 (9.97; 29.91)	26.73 (10.33; 32.18)	0.184
IL-8	12.84 (9.41; 21.21)	12.04 (8.26; 20.21)	0.544
IL-9	8 (3.5; 13.6)	11.86 (3.84; 13.6)	0.165
IL-12p40	45.83 (37.21;52.7)	47.55 (42.36; 54.42)	0.156
IL-12p70	16.24 (6.15; 23.71)	21.86 (7.49; 26.47)	0.019 *
IL-15	9.34 (6.26; 11.71)	10.83 (7.49; 12.3)	0.092
IL-17A	8.74 (4.29; 13.73)	12.23 (4.61; 15.59)	0.083
IFN-α2	52.11 (17.59; 90.62)	83.26 (23.65; 96.42)	0.175
IFN-γ	17.21 (9.97; 23.01)	19.39 (11.84; 23.73)	0.106
TNF-α	23.85 (17.98; 31.16)	25.20 (20.67; 29.63)	0.656
TNF-β	23.67 (6.00; 30.15)	25.53 (6.90; 35.62)	0.291
Anti-inflammatory cytokines			
IL-1RA	49.19 (38.55; 69.55)	46.02 (38.13; 60.68)	0.286
IL-4	122.08 (77.86; 160.49)	136.34 (81.48; 170.1)	0.189
IL-10	14.04 (7.85; 26.41)	23.44 (9.05; 27.89)	0.087
IL-13	19.47 (13.52; 24.02)	20.24 (13.75; 24.02)	0.479
TGF-α	4.67 (3.7; 6.94)	4.42 (3.44; 5.88)	0.181

Note: Data are presented as a median (lower quartile; upper quartile); groups were compared by the Mann–Whitney *U* test; *: statistically significant differences.

## Data Availability

The datasets are available on reasonable request to Svetlana A. Ivanova (ivanovaniipz@gmail.com), after approval from the Board of Directors of the Mental Health Research Institute, in accordance with local guidelines and regulations.

## Supplementary Materials

The following are available online at https://www.mdpi.com/article/10.3390/life12121972/s1, Table S1: Cytokine levels in the serum of patients with schizophrenia depending on disease duration; Table S2: Cytokine levels in the serum of patients with schizophrenia depending on the onset of disease.
