# Supplementary material for: Cytokines as Potential Biomarkers of Clinical Characteristics of Schizophrenia
Source: Life (Basel). 2022 Nov 25;12(12):1972. doi: 10.3390/life12121972 (PMC9784438; doi:10.3390/life12121972)
Supplement: Supplementary file 1 [file life-12-01972-s001.zip › life-1997055-supplementary.pdf]

Table S1. Cytokine levels in serum of patients with schizophrenia depending on duration of disease.

Parameter	≤5 Years (n = 52)	5–10 Years (n = 40)	≥10 Years (n = 144)	*p*-Value
		Pro-inflammatory cytokines		
IL-1α	63.16 (52.72; 100.85)	84.60 (54.95; 113.59)	81.16 (52.24; 105.10)	0.483
IL-1β	2.85 (2.21; 4.13)	3.84 (2.49; 4.83)	3.60 (2.35; 4.65)	0.162
IL-2	5.78 (4.67; 7.04)	6.21 (4.67; 7.52)	6.17 (4.73; 7.12)	0.821
IL-3	2.88 (1.86; 3.55)	2.44 (1.32; 3.32)	2.35 (1.26; 3.31)	0.118
IL-5	2.55 (1.73; 4.52)	3.06 (1.81; 4.64)	3.69 (2.08; 4.40)	0.458
IL-6	8.01 (4.65; 15.11)	9.86 (4.78; 15.93)	11.52 (5.86; 15.71)	0.309
IL-7	11.11 (9.66; 27.55(	13.29 (10.02; 28.94(	14.49 (9.63; 29.91)	0.293
IL-8	11.59 (8.55; 17.45)	11.64 (8.30; 25.35(	13.89 (9.93; 20.66)	0.230
IL-9	4.10 (2.76; 11.86)	6.68 (3.38; 13.60)	7.89 (3.47; 13.16)	0.307
IL-12p40	45.13 (37.16; 58.76)	44.52 (37.25; 52.70)	46.16 (39.68; 52.70)	0.738
IL-12p70	8.42 6.23; 55.56)	10.18 (6.14; 22.87)	11.79 (6.77; 24.98)	0.452
IL-15	8.34 (5.42; 9.91)	9.32 (6.37; 11.53)	9.52 (6.44; 12.01)	0.103
IL-17A	5.34(3.96; 15.03)	7.91 (4.05; 14.10(	10.54 (4.40; 14.85)	0.390
IFN-α2	26.81 (15.83; 81.77)	40.48 (16.14; 90.26)	45.05 (17.62; 91.71)	0.177
IFN-γ	12.83 (9.83; 23.40)	16.57 (10.03;22.11)	15.70 (10.12; 23.73)	0.630
TNF-α	21.13 (16.29; 26.66)	20.83 (14.66; 26.65(	24.95 (18.13; 30.02)	0.050
TNF-β	8.18 (4.07; 31.06)	16.06 (6.01; 30.73)	23.67 (6.45; 30.15)	0.270
		Anti-inflammatory cytokines		
IL-1RA	46.85 (38.50;57.16)	43.09 (38.42; 56.21)	49.71 (39.21; 70.71)	0.120
IL-4	86.84 (74.13; 151.59)	105.06 (71.59; 164.70)	124.36 (77.22; 157.25)	0.347
IL-10	10.13 (7.15; 22.39)*	12.91 (7.49; 26.23)	16.98 (8.31; 25.67)*	0.047*
IL-13	15.81 (12.23; 24.02)	16.84 (12.43; 23.65)	19.47 (13.75; 24.02)	0.323
TGF-α	5.05 (4.23; 6.97)	4.64 (4.05; 7.74)	5.15 (3.73; 7.31)	0.946

Note: Statistically significant difference according to the Kruskal-Wallis test; * – statistically significant differences between columns according to the Mann-Whitney test with Bonferroni correction for the three groups of comparisons ($p = 0.042$).

Table S2. Cytokine levels in serum of patients with schizophrenia depending on onset of disease.

**Parameter**	**≤18 Years (n = 27)**	**>18 Years (n = 209)**	***p*-Value**
Pro-inflammatory cytokines			
IL-1α	78.80 (54.67; 111.89)	70.10 (52.40; 105.10)	0.660
IL-1β	3.03 (2.49; 4.65)	3.30 (2.33; 4.48)	0.640
IL-2	6.44 (5.41; 7.57)	5.99 (4.67; 7.12)	0.243
IL-3	2.81 (1.26; 4.01)	2.55 (1.31; 3.32)	0.447
IL-5	3.72 (2.24; 4.13)	3.37 (1.97; 4.47)	0.975
IL-6	13.05 (4.60; 16.67)	10.34 (5.50; 15.33)	0.588
IL-7	14.55 (10.40; 28.35)	12.92 (9.62; 29.91)	0.857
IL-8	15.60 (9.00; 20.21)	12.84 (9.26; 20.03)	0.596
IL-9	4.56 (3.17; 13.16)	5.12 (3.32; 13.16)	0.999
IL-12p40	46.93 (41.29; 52.70)	45.83 (38.55; 53.29)	0.811
IL-12p70	9.69 (6.87; 23.71)	9.54 (6.72; 24.40)	0.916
IL-15	9.05 (6.82; 11.71)	9.05 (6.17; 11.71)	0.815
IL-17A	6.47 (4.31; 14.10)	6.68 (4.32; 14.85)	0.882
IFN-α2	46.99 (18.55; 90.62)	31.55 (17.23; 90.62)	0.715
IFN-γ	15.75 (10.46; 21.56)	14.60 (10.03; 23.01)	0.725
TNF-α	22.79 (19.30; 26.81)	22.95 (16.36; 29.43)	0.873
TNF-β	25.53 (6.01; 37.42)	20.86 (6.00; 29.23)	0.364
Anti-inflammatory cytokines			
IL-1RA	41.77 (36.21; 68.75)	47.08 (39.71; 63.58)	0.422
IL-4	117.11 (85.19; 170.10)	107.17 (74.85; 159.29)	0.265
IL-10	13.98 (8.00; 27.15)	12.25 (7.97; 25.67)	0.557
IL-13	19.47 (12.97; 23.27)	18.34 (13.18; 24.02)	0.786
TGF-α	5.25 (3.56; 6.84)	5.06 (4.00; 7.23)	0.550

Note: Data is presented as median (lower quartile; upper quartile); Comparisons between groups were performed using the Mann–Whitney U test.
